# LINC01004-SPI1 axis-activated SIGLEC9 in tumor-associated macrophages induces radioresistance and the formation of immunosuppressive tumor microenvironment in esophageal squamous cell carcinoma

**DOI:** 10.1007/s00262-022-03364-5

**Published:** 2023-01-23

**Authors:** Fen Zhao, Hui Tian, Yungang Wang, Jianbo Zhang, Fang Liu, Lei Fu

**Affiliations:** 1 Department of Radiation Oncology, Shandong Cancer Hospital and Institute, Shandong First Medical University and Shandong Academy of Medical Sciences, Jinan, 250117 Shandong People’s Republic of China; 2 Department of Radiation Oncology, Shandong Cancer Hospital and Institute, Shandong University, Jinan, 250117 Shandong People’s Republic of China; 3 Department of Radiation Oncology, Qilu Hospital of Shandong University, Jinan, 250012 Shandong People’s Republic of China; 4 Departments of Pathology, Shandong Cancer Hospital and Institute, Shandong First Medical University and Shandong Academy of Medical Sciences, Jinan, 250117 Shandong People’s Republic of China; 5 Department of Imaging, Shandong Medical College, Jinan, 250002 Shandong People’s Republic of China

**Keywords:** SIGLEC9, MUC1, Tumor-associated macrophages, Esophageal squamous cell carcinoma, Radioresistance

## Abstract

Radioresistance and immunosuppression remain the major obstacles in the anti-cancer treatments. This work studies the functions of sialic acid binding Ig like lectin 9 (SIGLEC9) and its related molecules in radioresistance and immunosuppression in esophageal squamous cell carcinoma (ESCC). The single-cell analysis showed that SIGLEC9 was mainly expressed on tumor-associated macrophages (TAMs). Monocytes-derived macrophages were co-cultured with ESCC cells and subjected to radiotherapy. High or low doses of radiotherapy induced SIGLEC9 upregulation and M2 polarization of TAMs. Artificial inhibition of SIGLEC9 in TAMs suppressed the radioresistance and immunosuppressive tumor microenvironment (TME) in the co-cultured ESCC cells. Upstream molecules of SIGLEC9 were predicted via bioinformatics. LINC01004 recruited Spi-1 proto-oncogene (SPI1) in nucleus of TAMs to induce transcriptional activation of SIGLEC9. SIGLEC9 interacted with mucin 1 (MUC1). MUC1 overexpression in ESCCs induced M2 skewing of TAMs, enhanced radioresistance and immunosuppression, and promoted nuclear translocation of β-catenin to suppress radiotherapy-induced ferroptosis of ESCC cells. These effects were blocked upon SIGLEC9 suppression. In vitro results were reproduced in the animal models with xenograft tumors. Taken together, this study demonstrates that the LINC01004-SPI1 axis-activated SIGLEC9 in TAMs induces radioresistance and the formation of immunosuppressive TME in ESCC.

**Supplementary Information:**

The online version contains supplementary material available at 10.1007/s00262-022-03364-5.

## Introduction

Esophageal cancer represents the seventh most prevalent cancer and the sixth leading cause of cancer-related mortality worldwide, which accounts for one in every 18 cancer deaths in 2020 [1]. Esophageal squamous cell carcinoma (ESCC), which initiates via malignant transformation of epithelial cells, accounts for over 90% of all esophageal cancers and represents one of the most aggressive forms of squamous cell carcinomas with the 5-year survival rate lower than 20% [2]. Post-surgery neoadjuvant chemoradiotherapy remains the major regimen for locally advanced ESCC, but the therapeutic benefit is unsatisfactory [3] mainly due to the radioresistance [4]. The formation of immunosuppressive tumor microenvironment (TME) after radiotherapy further enhances the radioresistance and triggers tumor cell dissemination [5]. A previous publication demonstrates that several immune-suppressive cells are enriched in TME of ESCC, including regulatory T cells, exhausted T cells, natural killer cells, tolerogenic dendritic cells, and M2 macrophages [6]. Therefore, attempts to re-build the immunosuppressive TME are hopeful to overcome radioresistance and improve the efficacy of radiotherapy.

By querying bioinformatics tools and systems, we obtained sialic acid binding Ig like lectin 9 (SIGLEC9) as a significantly elevated molecule mainly expressed on macrophages in ESCC tissues after radiotherapy. SIGLEC9 belongs to the SIGLEC family of pattern-recognition receptors on immune cells that can bind to a range of sialoglycan ligands, leading to evasion of innate immune responses and tumor progression [7, 8]. Infiltration of SIGLEC9-expressing immune cells has been associated with immunosuppression and tumor development [9]. Tumor-associated macrophages (TAMs), which are mainly derived from circulating monocytes to tumors, are a large population of immune cells in the TME [10]. TAMs are generally classified into classical activated M1 phenotype and alternatively activated M2 type: the former type TAMs exert anti-tumor effects, whereas the latter type TAMs help immune evasion and promote tumor development [11]. Of note, high-dose radiotherapy usually leads to M2 skewing of TAMs, which is also related to radioresistance [12]. Considering that SIGLEC9 in TAMs has been reported to reduce inflammatory programs and increase the levels of M2-type markers [13], we postulated that aberrant expression of SIGLEC9 is potentially linked to immunosuppressive TME and radioresistance in ESCC.

Mucins are a class of high molecular-weight epithelial glycoproteins which help maintain tissue homeostasis by protecting epithelial barriers against environmental insults; however, aberrant overexpression and glycosylation of mucins are closely linked to a variety of oncogenic events from tumor initiation to development [14]. Mucin 1 (MUC1) is the first-identified mucin detected in pancreatic cancer, and its upregulation was then found in several types of solid cancers, leaving it as a candidate target for cancer therapy [15]. In cancer, the abnormal O-linked glycosylation of MUC1 can affect the interaction between MUC1 and lectins of the immune system [16]. Intriguingly, MUC1 expressed on cancer cells has been reported to engage SIGLEC9 to modulate the TME [17]. The interaction of MUC1 with SIGLEC9 in radioresistance and TME formation in ESCC attracted our interests.

Ferroptosis, a new form of programmed cell death characterized by iron-dependent and lethal lipid peroxidation accumulation, can be triggered by radiotherapy-induced accumulation of reactive oxygen species (ROS) [18, 19]. Glutathione peroxidase 4 (GPX4) is an anti-ferroptosis protein that induces the reduction reaction of lipid peroxide and balances the redox reaction [19]. Interestingly, MUC1 has been reported as a suppressor of ferroptosis [20]. It is also noteworthy that the MUC1-SIGLEC9 interaction can modulate nuclear translocation of β-catenin and cell growth [21]. Moreover, β-catenin has been reported to upregulate GPX4 expression [22], therefore possibly playing a role in protecting cells from ferroptosis. Taken together, this study aims to investigate the function of SIGLEC9 and its interaction with MUC1 in TAM polarization, TME and radioresistance in ESCC. Moreover, the potential mechanism responsible for aberrant SIGLEG9 expression in ESCC was explored.

## Materials and methods

### Clinical samples

Tumor tissue samples were collected from 40 patients with ESCC treated at our hospital from April 2019 to May 2021. All patients were free of other malignances, among which 20 did not receive radiotherapy (RT^–^), and the rest 20 received radiotherapy (RT^+^; with accumulative doses exceeding 40 Gy) before surgery. Moreover, the RT^+^ patients were further divided into the Poor response group (with no tumor regression or even tumor expansion; *n* = 6) and the Response group (with significant tumor regression; *n* = 14). Human peripheral blood samples were collected from 10 healthy donors via venipuncture.

### RNA isolation and quantification

Total RNA from cells was isolated using the TRIzol reagent (Invitrogen) and reverse-transcribed to cDNA by the PrimeScript™ RT reagent Kit with gDNA Eraser (Takara Holdings Inc., Kyoto, Japan). The quantitative polymerase chain reaction (qPCR) analysis was then conducted using TB Green® Premix Ex Taq™ (Takara) on the CFX96 Real-Time PCR Detection System (Bio-Rad, Inc., Hercules, CA, USA). Relative expression of genes was analyzed by the 2^−ΔΔCT^ method with glyceraldehyde-3-phosphate dehydrogenase (GAPDH) as the internal control. The primers are presented in Table S1.

### Statistical analysis

All experimental data were collected from at least three independent experiments. Statistical analyses were performed by GraphPad Prism 8.0 (GraphPad Software Inc., CA, USA). The Shapiro–Wilk test was performed to check the normality of data. Differences between groups were analyzed by the unpaired *t* test, or by two-way analysis of variance (ANOVA) if over two groups were involved. The post hoc analyses were performed by Dunnett's, Sidak's, or Tukey’s multiple comparisons test. *p < 0.05 was considered to present significant difference.

The methodologies of (Immunohistochemistry (IHC), Double-label immunofluorescence, Cells, Cell treatment, Flow cytometry, Cell necrosis detection, 5-ethynyl-2’-deoxyuridine (EdU) labeling assay, Transwell assays, Enzyme-linked immunosorbent assay (ELISA) and oxidative stress examination, Western blot (WB) analysis, Chromatin immunoprecipitation (ChIP), Luciferase reporter assay, RNA immunoprecipitation (RIP), Co-immunoprecipitation (Co-IP), and Animals) were included in Electronic Supplementary Material (Supplementary file 1).

## Results

### SIGLEC9 is upregulated in ESCC tissues with radiotherapy

**Fig. 1:**
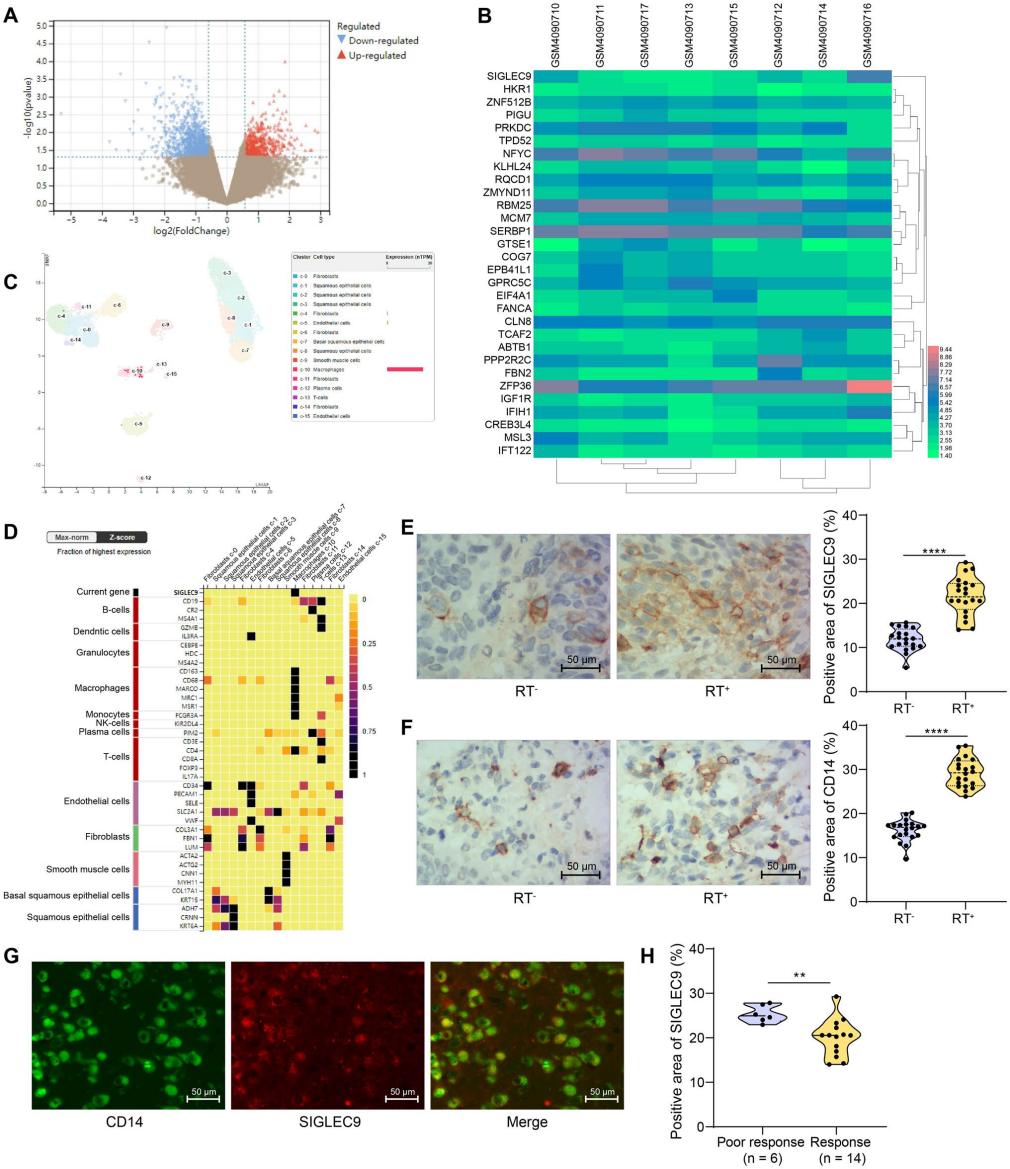
SIGLEC9 is upregulated in ESCC tissues with radiotherapy. **a** genes with differential expression in ESCC before and after radiotherapy analyzed by the GEO dataset GSE137867; **b** SIGLEC9 with significant upregulation in ESCC tissues after radiotherapy according to the dataset analysis; **c**, SIGLEC9 expression in esophagus analyzed by single-cell analysis; **d**, correlation of SIGLEC9 expression with cellular markers in esophagus tissue; **e**, expression of SIGLEC9 in clinically collected RT^+^ and RT^–^ ESCC tissues analyzed by IHC; **f**, infiltration of CD14^+^ TAMs in ESCC tissues analyzed by IHC; **g**, co-localization of SIGLEC9 and CD14 in RT^+^ ESCC tissues analyzed by double-label immunofluorescence; **h**, expression of SIGLEC9 in patients with or without significant response to radiotherapy. The unpaired *t* test (**e**, **f**, **h**) was used to compare normally distributed data between two groups. **p < 0.01, ****p < 0.0001

By analyzing the GEO dataset GSE137867 (https://www.ncbi.nlm.nih.gov/geo/query/acc.cgi?acc=GSE137867) (Fig. 1a), we identified SIGLEC9 (11729874_at) as a significantly elevated gene in ESCC tissues after radiotherapy (Fig. 1b). Thereafter, we performed single-cell analysis in the HUMAN PROTEIN ATLAS (HPA) system (https://www.proteinatlas.org/). In esophagus, SIGLEC9 is mainly expressed on macrophages (Fig. 1c). This is partly accorded with the report that SIGLEC9 is mainly expressed on infiltrating immune cells during tumor progression [9]. Moreover, further analysis in the system showed that the SIGLEC9 expression was linked to the expression of macrophage markers such as CD163 and CD68 in esophagus (Fig. 1d).

According to IHC results, SIGLEC9 expression was significantly increased in the tumor tissues or patients with radiotherapy (RT^+^) compared to those without (RT^–^) (Fig. 1e). Moreover, the radiotherapy led to an elevation in the expression of CD14 (Fig. 1f), a TAM marker [23]. The double-label immunofluorescence confirmed a co-localization of SIGLEC9 and CD14 in the RT^+^ ESCC tissues (Fig. 1g). Moreover, elevated SIGLEC9 expression was detected in patients with poor response to radiotherapy (Fig. 1h).

## Radiotherapy induces phenotype change of TAMs

Immune cells including TAMs can affect the efficacy of anti-cancer treatments such as chemotherapy, radiotherapy, and kinase inhibitor treatments [24]. We induced macrophages and had them co-cultured with ESCC cells (TE-1 and KYSE-30) at 1:3. During the 5-day co-culture, the cells were exposed to different doses of irradiation (0, 0.5, 1, 2, 4, 6 and 8 Gy) (Fig S1A). The co-cultured (Co^+^) TAMs and ESCC cells were collected for subsequent analyses. Macrophages or ESCC cells cultured separately (Co^–^) were set as controls to analyze the direct effect of radiotherapy alone.

As shown in Fig S1B, compared to the Co^–^ macrophages, the Co^+^ TAMs showed a polarization trend toward M2 phenotype (CD163^+^). The Co^–^ macrophages switched to M1 phenotype (CD11C^+^) after radiotherapy in a discontinuous dose-dependent manner. Intriguingly, the Co^+^ TAMs showed more special phenotype alteration after radiotherapy. In response to low (< 1 Gy) or high doses (> 4 Gy) of irradiation, they tended to switch to the M2 phenotype (CD163^+^); however, when exposed to moderate doses (1–4 Gy) of irradiation, more Co^+^ TAMs skewed to the M1 phenotype (CD11C^+^). The qPCR analysis concerning the expression of macrophage-related factors showed similar results (Fig S1C-D). The Co^–^ macrophages showed increased expression of M1 cytokines including tumor necrosis factor-α (TNF-α) and interleukin (IL)-12 whereas decreased expression of M2-related IL-10 and PD-L1. The low-dose (< 1 Gy) or high-doses (> 4 Gy) of irradiation increased the expression of M2 cytokines whereas decreased M1 cytokines in the Co^+^ TAMs; However, the moderate doses (1–4 Gy) of irradiation promoted the expression of M1 cytokines. The qPCR analysis showed that the SIGLEC9 expression was gradually decreased in Co^–^ macrophages after irradiation. Of note, the SIGLEC9 expression in Co^+^ TAMs was increased when exposed to low or high doses of irradiation but decreased when exposed to moderate doses of irradiation (Fig S1E).

### TAMs mediate radioresistance of ESCCs and the formation of immunosuppressive TME

**Fig. 2:**
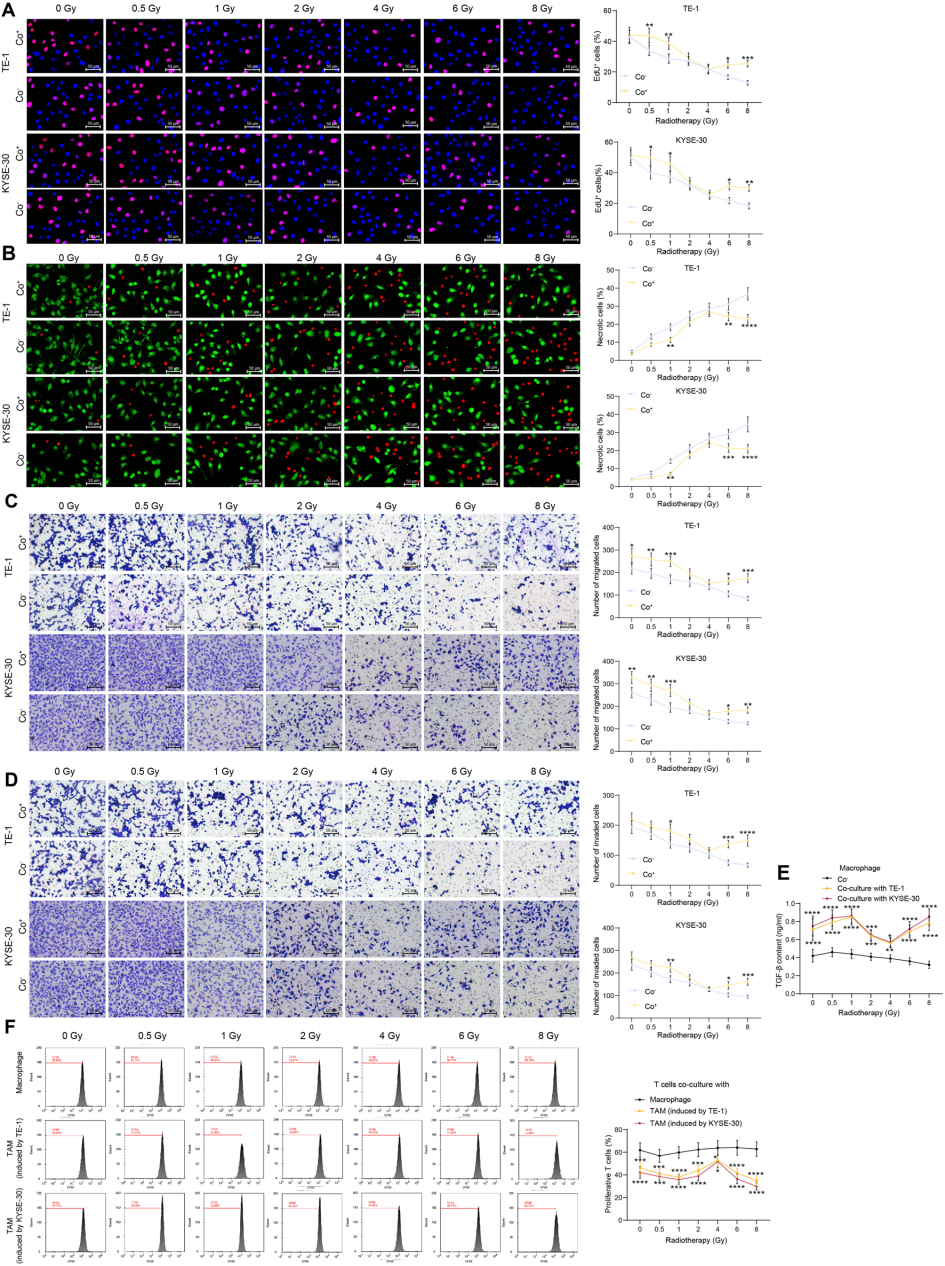
TAMs mediate radioresistance of ESCCs and formation of immunosuppressive TME. **a**, DNA damage sensitivity of ESCC cells to radiotherapy examined by EdU labeling; **b**, necrosis of ESCC cells analyzed by Calcein AM and 7-AAD staining; **c-d**, migration (**c**) and invasion (**d**) of ESCCs analyzed by Transwell assays; **e**, release of immunosuppression marker TGF-β by macrophages analyzed by ELISA kits; **f**, proliferation of active T cells co-cultured with macrophages analyzed by CFSE staining. Differences of the normally distributed data between groups were analyzed by two-way ANOVA (**a–e**). Significance of difference was analyzed by Sidak's multiple comparisons test (**a–d**) or Dunnett's multiple comparisons test (**e**–**f**). *p < 0.05, **p < 0.01, ***p < 0.001, ****p < 0.0001

The response of ESCC cells to radiotherapy was then analyzed. The EdU assay showed that the radiotherapy induced DNA damage in ESCC cells to various degrees whether they were co-cultured (Co^+^) with macrophages or not (Co^–^). The Co^–^ ESCC cells showed a dose-independent impairment of DNA synthesis in response to radiotherapy. However, the Co^+^ ESCC cells showed certain degrees of radioresistance when exposed to low or high doses of irradiation (Fig. 2a). The alive cells (green) and necrotic cells (red) were stained with Calcein AM and 7-AAD, respectively. The radiotherapy induced cell necrosis. This pro-necrotic role of irradiation showed a dose-dependent manner in Co^–^ ESCC cells. However, the Co^+^ ESCC cells showed resistance to necrosis upon exposure to low or high doses of irradiation (Fig. 2b). Moreover, the radiotherapy significantly suppressed the migration and invasiveness of ESCC cells. However, the suppressive effect of radiotherapy on the mobility of Co^+^ ESCC cells was reduced when low or high irradiation doses were administrated (Fig. 2c, d).

In addition to directly influencing tumor cells, TAMs can release multiple signals such as TGF-β to affect infiltration of T cells, therefore suppressing the anti-tumor immune response [25]. Here, we found by ELISA that the release of TGF-β by Co^–^ macrophages cells was not substantially affected by radiotherapy. For Co^+^ TAMs, however, the TGF-β release was reduced by moderate doses but increased by low or high doses of irradiation (Fig. 2e). Moreover, the irradiation-exposed macrophages were harvested and co-cultured with CFSE-labeled active T cells (1:1). The flow cytometry showed that the Co^–^ macrophages did not significantly affect the proliferation of T cells. After exposure to moderate doses of irradiation, the Co^+^ TAMs induced proliferation of active T cells; however, the low or high doses of irradiation-stimulated Co^+^ TAMs significantly blocked the T cell proliferation (Fig. 2f).

### SIGLEC9 participates in the radioresistance and immunosuppression mediated by TAMs

The focus was then shifted to verifying the potential involvement of SIGLEC9 in the events mediated by TAMs. Considering that patients are generally subjected to radiotherapy for multiple times, therefore, a high-dose irradiation exposure was used as the scheme for subsequent in vitro experiments. The macrophages were pre-treated with the SIGLEC9 neutralizing monoclonal antibody with the isotype IgG as control, followed by co-culture with cancer cells and exposure to high-dose (8 Gy) of irradiation for subsequent analysis.

**Fig. 3:**
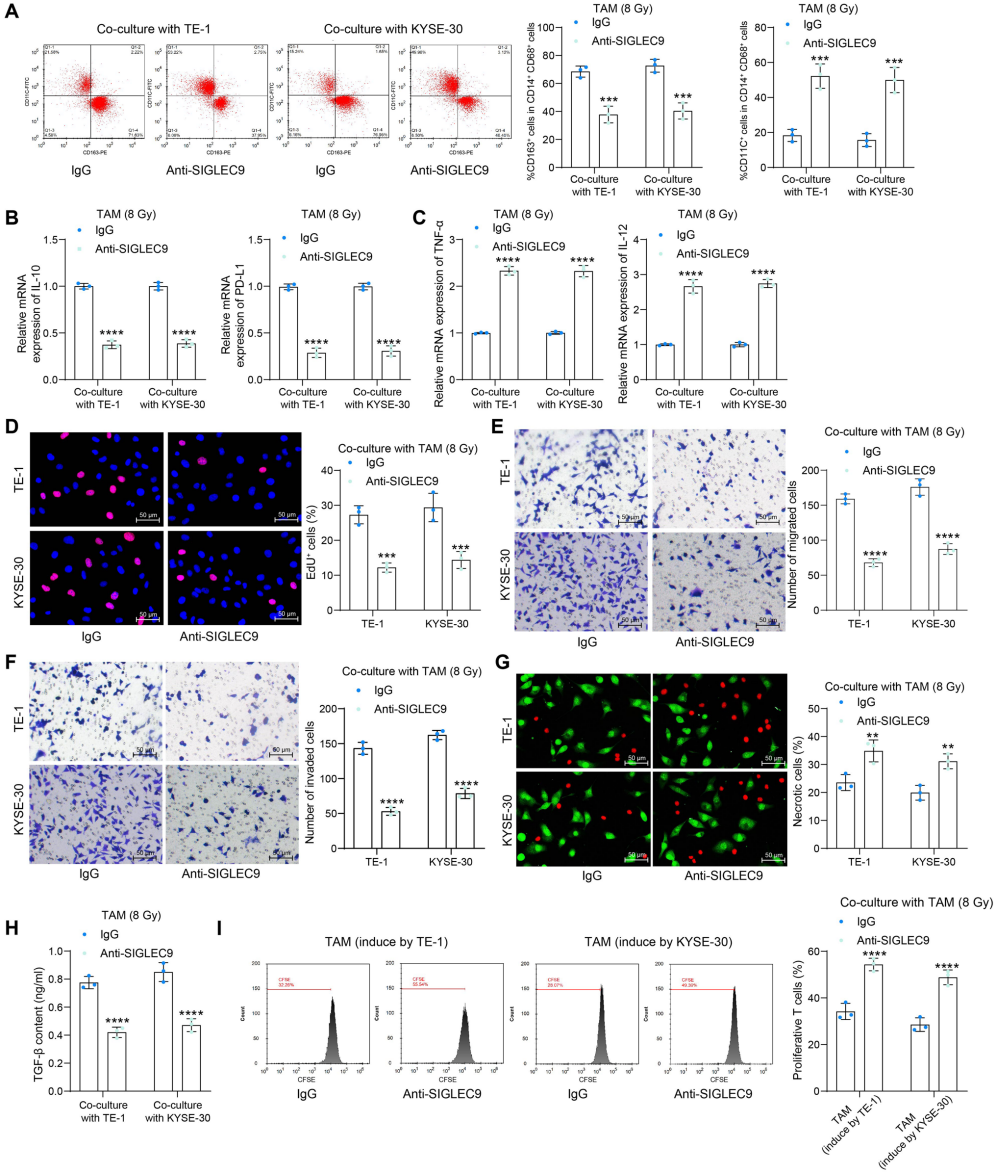
SIGLEC9 participates in the radioresistance and immunosuppression mediated by TAMs. **a**, phenotype change of TAMs with SIGLEC9 antibody treatment analyzed by flow cytometry; **b**, **c**, expression of M2 phenotype markers IL-10 and PD-L1 (**b**) and M1 phenotype markers TNF-α and IL-12 (**c**) in TAMs with SIGLEC9 antibody treatment analyzed by qPCR analysis; **d**, DNA damage in ESCC cells examined by EdU labeling; **e**–**f**, migration (**e**) and invasion (**f**) of ESCCs analyzed by Transwell assays; **g**, necrosis of ESCC cells analyzed by Calcein AM and 7-AAD staining; **h**, release of immunosuppression marker TGF-β by TAMs analyzed by ELISA kits; **i**, proliferation of active T cells co-cultured with TAMs analyzed by CFSE staining. Differences of the normally distributed data between groups were analyzed by two-way ANOVA (**a**–**i**). Significance of difference was analyzed by Sidak's multiple comparisons test (**a–i**). **p < 0.01, ***p < 0.001, ****p < 0.0001

Of note, the SIGLEC9 blockade suppressed high-dose irradiation-induced M2 polarization of TAMs (Fig. 3a). The qPCR analysis also revealed that the SIGLEC9 neutralizing antibody in TAMs decreased the M2 cytokines IL-10 and PD-L1 but increased the M1 cytokines TNF-α and IL-12 (Fig. 3b, c).

The radioresistance of ESCCs stimulated by the TAMs was reduced upon SIGLEC9 blockade, as manifested by significantly suppressed DNA synthesis capacity, migration, and invasion of the ESCC cells (Fig. 3d–f), along with increased cell necrosis (Fig. 3g). Moreover, upon SIGLEC9 blockade, the TGF-β release by TAMs (Fig. 3h) and the suppressive effect of TAMs on T cell proliferation (Fig. 3i) were reduced.

### SPI1 is upregulated in high-dose irradiation-exposed TAMs and activates SIGLEC9 transcription

**Fig. 4:**
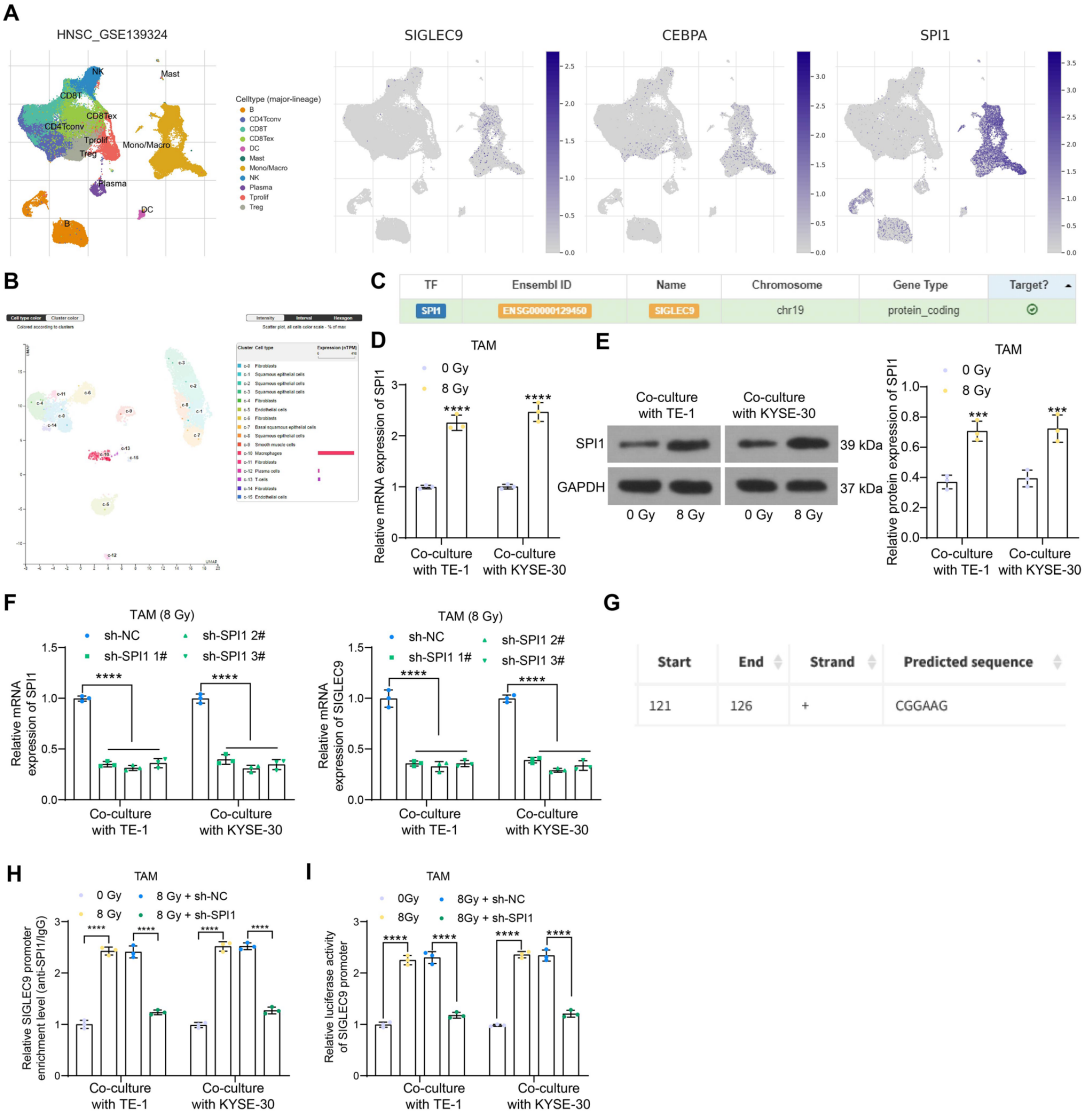
SPI1 is upregulated in high-dose irradiation-exposed TAMs and activates SIGLEC9 transcription. **a**, expression of transcription factors in different types of immune cells in HNSC analyzed by single-cell analysis; **b**, expression of CEBPA and SPI1 in esophagus analyzed by single-cell analysis; **c**, SPI1 predicted as a candidate upstream regulator of SIGLEC9; **d-e**, mRNA and protein expression of SPI1 in high-dose irradiation-exposed TAMs analyzed by qPCR (**d**) and WB (**e**) analyses; **f**, SPI1 and SIGLEC9 mRNA in TAMs pre-transfected with sh-SPI1 analyzed by qPCR analysis; **g**, putative binding site of SPI1 to SIGLEC9 promoter; **h**, binding of SIGLEC9 promoter with SPI1 in TAMs upon high-dose irradiation treatment and sh-SPI1 transfection analyzed by ChIP-qPCR assay; **i**, transcription activity of SIGLEC9 promoter in TAMs upon high-dose irradiation treatment and sh-SPI1 transfection analyzed by luciferase assay. Differences of the normally distributed data between groups were analyzed by two-way ANOVA (**d**, **e**, **f**, **h** and **i**). Significance of difference was analyzed by Sidak's multiple comparisons test (**d** and **e**), Dunnett's multiple comparisons test (**f**) and Tukey's multiple comparisons test (**h** and **i**). ***p < 0.001, ****p < 0.0001

To unravel the mechanism responsible for SIGLEC9 upregulation in high-dose irradiation-exposed TAMs, we queried transcription factors that can target SIGLEC9 in the human transcription factor database hTFtarget (http://bioinfo.life.hust.edu.cn/hTFtarget/#!/). The expression of the top 30 candidate transcription factors in immune cells in head and neck squamous carcinoma (HNSC, including ESCC) was analyzed via the TISCH database (http://tisch.comp-genomics.org/home/) to verify if the transcription factors have similar expression profiling with SIGLEC9. In the database, SIGLEG9 still showed a high abundance in macrophages. Among the 30 candidate factors, only CEBPA and SPI1 were similarly highly abundant in the infiltrating macrophages in HNSC (Fig. 4a). In the HPA system, only SPI1 was highly expressed on macrophages in esophagus (Fig. 4b). Therefore, we postulated that SPI1 possibly regulates SIGLEC9 transcription in macrophages (Fig. 4c). Of note, the subsequent qPCR and WB analyses revealed that the high-dose irradiation induced SPI1 expression in TAMs (Fig. 4d, e). Pre-transfection of the SPI1 shRNAs suppressed the SPI1 expression in macrophages, along with reduced SIGLEC9 expression (Fig. 4f). The sh-SPI1 2# with the best suppressive effect was selected for subsequent use. We obtained the binding region of SPI1 to SIGLEC9 promoter from Jaspar (http://jaspar.genereg.net/) (Fig. 4g). The ChIP-qPCR analysis showed that the high-dose irradiation stimulated the binding between SPI1 and the promoter region of SIGLEC9 containing the putative binding site, whereas this binding relationship was weakened by sh-SPI1 (Fig. 4h). Moreover, the luciferase assay revealed that the high-dose irradiation-induced SPI1 elevation triggered transcriptional activation of the SIGLEC9 promoter, which was suppressed upon sh-SPI1 transfection in cells (Fig. 4i).

### LINC01004 is responsible for the transcriptional activation of SIGLEC9 mediated by SPI1

**Fig. 5:**
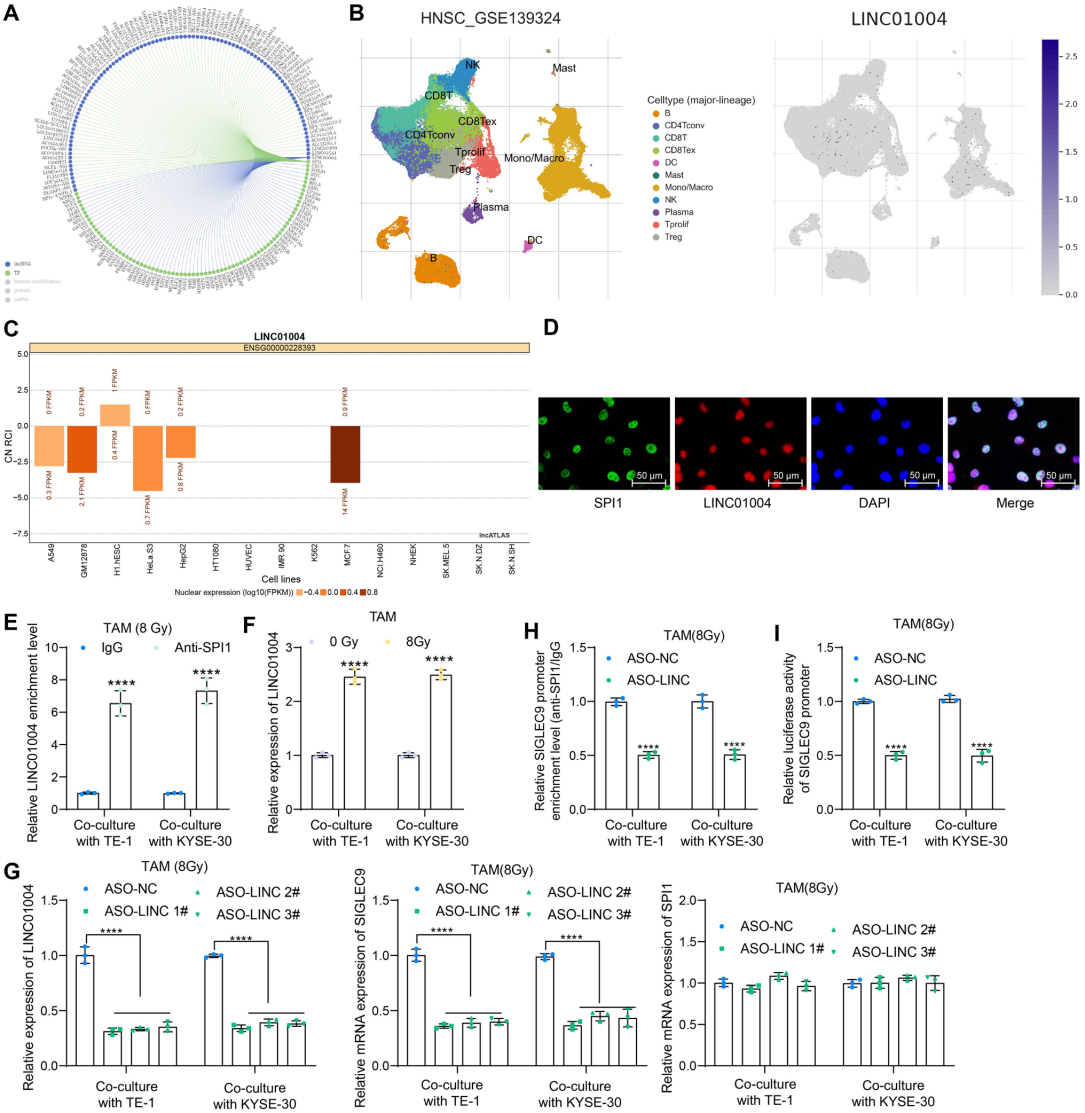
LINC01004 is responsible for the transcriptional activation of SIGLEC9 mediated by SPI1. **a**, lncRNAs that can interact with SPI1 predicted via bioinformatics; **b**, LINC01004 expression in different types of immune cells in HNSC analyzed by single-cell analysis; **c**, sub-cellular localization of LINC01004 in cells precited via bioinformatics; **d**, co-localization of SPI1 and LINC01004 in macrophages analyzed by fluorescence in situ hybridization of LINC01004 along with immunofluorescence staining of SPI1; **e**, binding of LINC01004 and SPI1 protein in macrophages analyzed by RIP assay; **f**, LINC01004 expression in high-dose irradiation-exposed TAMs analyzed by qPCR analysis; **g**, expression of LINC01004, SPI1 mRNA, and SIGLEC9 mRNA in TAMs transfected with ASO-LINC analyzed by qPCR analysis; **h**, binding of SPI1 with SIGLEC9 promoter in TAMs upon ASO-LINC transfection determined by the ChIP-qPCR assay; **i**, transcription activity of SIGLEC9 promoter in TAMs upon ASO-LINC transfection analyzed by luciferase assay. Differences of the normally distributed data between groups were analyzed by two-way ANOVA (**e**–**i**). Significance of difference was analyzed by Sidak's multiple comparisons test (**e**, **f**, **h** and **i**) and Dunnett's multiple comparisons test (**g**). ****p < 0.0001

Intriguingly, the regulation of SPI1 on the transcription of molecular substrates in several cancer cases has been reported to involve lncRNAs [26, 27]. We wondered if any lncRNA holds accountable for the SPI1-mediated SIGLEC9 activation in TAMs. Therefore, lncRNAs that can interact with SPI1 were explored via the RNA interactome database RNAInter (http://www.rnainter.org/search/) (Fig. 5a). Alike, the expression of the candidate lncRNAs in the immune cells in HNSC was queried in the TISCH database, and only LINC01004 was highly abundant in the macrophages (Fig. 5b). According to the LncATLAS database (https://lncatlas.crg.eu/), LINC01004 is mainly distributed in nucleus (Fig. 5c). We performed fluorescence in situ hybridization of LINC01004 (Cy3 fluorescence probe) along with the immunofluorescence staining of SPI1 and confirmed that the LINC01004 had co-localization with SPI1 in the nucleus of macrophages (Fig. 5d). The binding of SPI1 protein with LINC01004 was confirmed by the RIP assay (Fig. 5e). The qPCR analysis identified elevated LINC01004 expression in high-dose irradiation-exposed TAMs (Fig. 5f). In TAMs pre-transfected with LINC01004 ASOs, the LINC01004 and SIGLEC9 expression was significantly reduced; however, the SPI1 expression was not significantly altered (Fig. 5g). The most effective ASO-LINC 1# was selected for subsequent use. Silencing of LINC01004 reduced the enrichment of SPI1 at SIGLEC9 promoter (Fig. 5h) and reduced the transcriptional activation of SIGLEC9 (Fig. 5i).

### The LINC01004-SPI1 axis-mediated SIGLEC9 affects TAM-mediated radioresistance and immunosuppression in ESCC cells

**Fig. 6:**
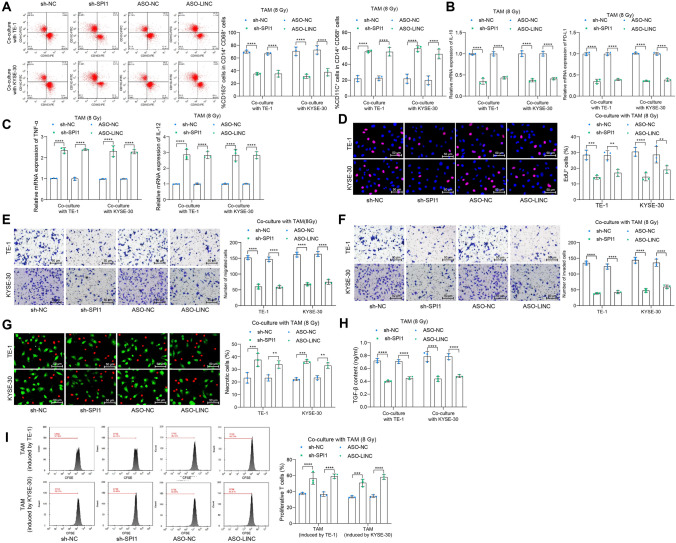
The LINC01004-SPI1 axis-mediated SIGLEC9 affects TAM-mediated radioresistance and immunosuppression in ESCC cells. **a**, phenotype change of TAMs with SPI1 shRNA or LINC01004 ASO transfection analyzed by flow cytometry; **b**, **c**, expression of M2 phenotype markers IL-10 and PD-L1 (**b**) and M1 phenotype markers TNF-α and IL-12 (**c**) in TAMs with SPI1 shRNA or LINC01004 ASO transfection analyzed by qPCR analysis; **d**, DNA damage in ESCC cells examined by EdU labeling; **e**–**f**, migration (**e**) and invasion (**f**) of ESCCs analyzed by Transwell assays; **g**, necrosis of ESCC cells analyzed by Calcein AM and 7-AAD staining; **h**, release of immunosuppression marker TGF-β by TAMs analyzed by ELISA kits; **i**, proliferation of active T cells co-cultured with TAMs analyzed by CFSE staining. Differences of the normally distributed data between groups were analyzed by two-way ANOVA (**a**–**i**). Significance of difference was analyzed by Sidak's multiple comparisons test (**a–i**). **p < 0.01, ***p < 0.001, ****p < 0.0001

The effects of LINC01004 ASO or SPI1 shRNA on the function of high-dose (8 Gy) irradiation-exposed TAMs were analyzed. As expected, blockade of the LINC01004-SPI1 axis and the subsequent SIGLEC9 inactivation led to M1 skewing of the irradiation-exposed TAMs (Fig. 6a), a decline in the expression of IL-10 and PD-L1 (Fig. 6b), and an increase in the expression of TNF-α and IL-12 (Fig. 6c). The ESCCs co-cultured with the shRNA or ASO-transfected TAMs were analyzed as well. It was found that upon the suppression of LINC01004 or SPI1 and the subsequent SIGLEC9 inhibition, the DNA synthesis ability, and the migration and invasion activities of cancer cells were significantly suppressed (Fig. 6d–f), along with increased necrosis rate of cells (Fig. 6g). Moreover, the shRNA or ASO treatments in TAMs also reduced TGF-β release (Fig. 6h) and restored the proliferation of the co-cultured active T cells (Fig. 6i).

### SIGLEC9 participates in MUC1-mediated re-education of TAMs

**Fig. 7:**
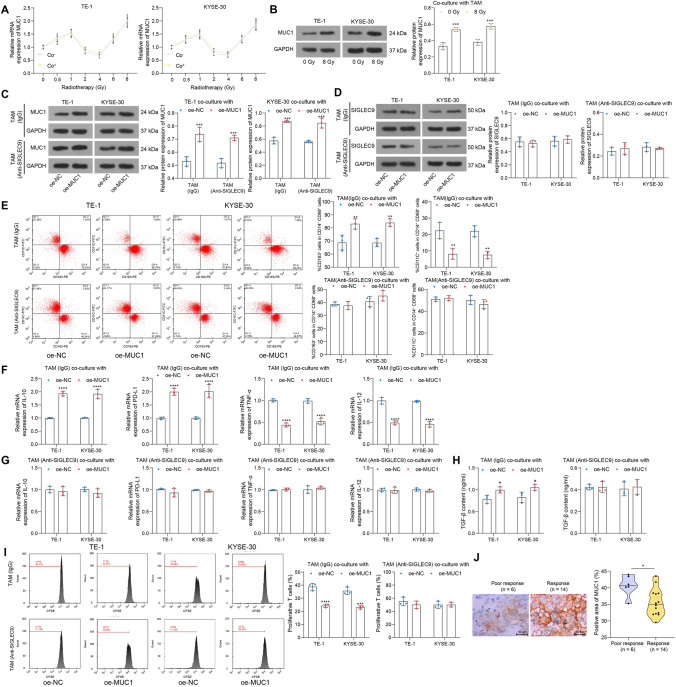
SIGLEC9 participates in MUC1-mediated re-education of TAMs. **a**, MUC1 mRNA expression in Co^–^ and Co^+^ ESCCs analyzed by qPCR analysis; **b**, MUC1 protein level in ESCC cells after high-dose (8 Gy) irradiation determined by WB analysis; **c**, MUC1 protein level in Co^+^ ESCCs exposed to high-dose (8 Gy) irradiation determined by WB analysis; **d**, SIGLEC9 protein level in TAMs co-cultured with ESCC cells transfected with oe-MUC1 or oe-NC; **e**, phenotype change of TAMs analyzed by flow cytometry; **f**, **g**, expression of M2 phenotype markers IL-10 and PD-L1 and M1 phenotype markers TNF-α and IL-12 in TAMs analyzed by qPCR analysis; **h**, release of immunosuppression marker TGF-β by TAMs analyzed by ELISA kits; **i**, proliferation of active T cells co-cultured with TAMs analyzed by CFSE staining; **j**, expression of MUC1 in patients with or without significant response to radiotherapy determined by IHC assay. Differences of the normally distributed data between groups were analyzed by the unpaired *t* test (**j**) or two-way ANOVA (**b**–**i**). Significance of difference was analyzed by Sidak's multiple comparisons test (**a**–**j**). *p < 0.05, **p < 0.01, ***p < 0.001, ****p < 0.0001

MUC1 on cancer cells has been reported to bind to SIGLEC9 on macrophages to re-educate the macrophages and help release factors linking to TME and cancer progression [17]. This may explain why the Co^–^ macrophages and Co^+^ TAMs showed distinct phenotype alteration upon radiotherapy. Importantly, we observed that the low dose (< 1 Gy) or high doses (> 4 Gy) of irradiation increased whereas the moderate doses suppressed MUC1 expression in ESCC cells, regardless they were co-cultured with macrophages or not (Fig. 7a). The MUC1 expression in cancer cells might only be affected by the cellular response to radiotherapy but not affected by TAM stimulation. Moreover, the MUC1 expression in ESCC cells was significantly increased when exposed to 8 Gy irradiation (Fig. 7b).

TAMs did not affect MUC1 expression, though, would MUC1 expression affect SIGLEC9 expression in TAMs? To examine this, we induced MUC1 overexpression in ESCC cells via transfection of DNA plasmids. The transfected cells were co-cultured with macrophages with or without SIGLEC9 antibody treatment and exposed to high-dose (8 Gy) irradiation. It was found that oe-MUC1 promoted MUC1 expression in ESCC cells but did not affect SIGLEC9 expression in the co-cultured TAMs, and artificial inhibition of SIGLEC9 in TAMs did not affect the MUC1 expression in the co-cultured ESCC cells as well (Fig. 7c, d). Intriguingly, when analyzed the TAM phenotype, we observed that oe-MUC1-transfected ESCC cells stimulated the M2 skewing of the co-cultured TAMs, but the polarization of TAMs was not affected by oe-MUC1 when SIGLEC9 was suppressed (Fig. 7e). At the molecular level, the expression of IL-10 and PD-L1 was increased, whereas the expression of TNF-α and IL-12 in TAMs was suppressed when they were co-cultured with ESCC cells overexpressing MUC1. Again, the effect of MUC1 on molecular changes was diminished when SIGLEC9 was suppressed in the TAMs (Fig. 7f-g). Upon oe-MUC1 stimulation, the TGF-β release by TAMs was increased and proliferation of T cells was decreased; however, the MUC1 overexpression condition no longer had effects on the TAMs where SIGLEC9 was blocked (Fig. 7h-i). This ample evidence suggests that the MUC1-mediated re-education of TAMs requires the participance of SIGLEC9. Meanwhile, elevated MUC1 expression was detected in patients with poor response to radiotherapy as well (Fig. 7).

### *MUC1 suppresses radiotherapy-induced ferroptosis of ESCC cells *via* SIGLEC9*

**Fig. 8:**
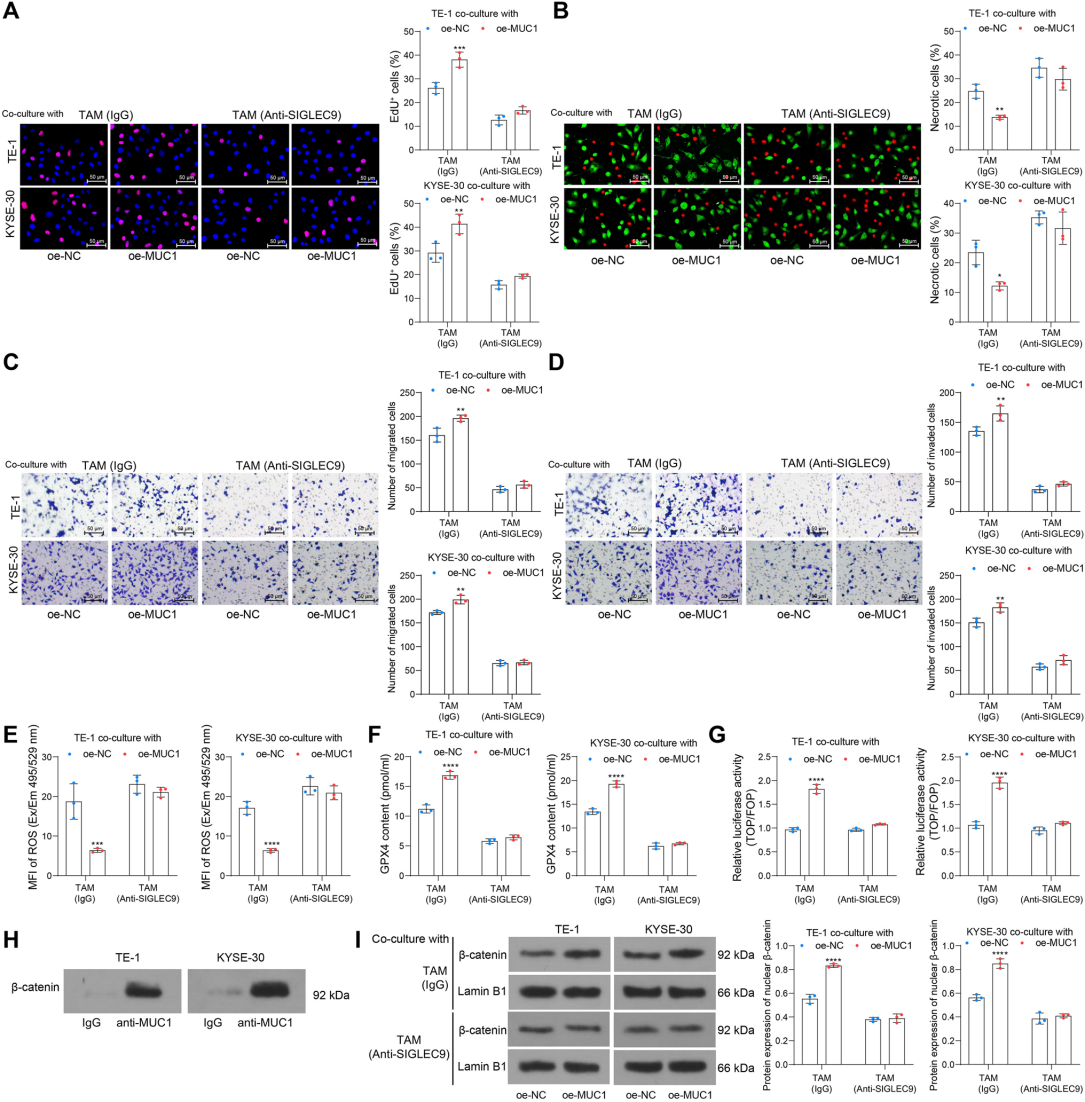
MUC1 suppresses radiotherapy-induced ferroptosis of ESCC cells via SIGLEC9. **b**, DNA damage in ESCC cells examined by EdU labeling; **b**, necrosis of ESCC cells analyzed by Calcein AM and 7-AAD staining; **c**, **d**, migration (**c**) and invasion (**d**) of ESCCs analyzed by Transwell assays; **e**, mean fluorescence intensity of ROS in each group of ESCC cells; **f**, GPX4 concentration in each group of ESCC cells analyzed by ELISA kits; **g**, β-catenin activity in ESCC cells analyzed by the TOP/FOP Flash assay; **h**, binding between MUC1 and β-catenin analyzed by the Co-IP assay; **i**, protein level of β-catenin in ESCC cells analyzed by WB analysis. Differences of the normally distributed data between groups were analyzed by two-way ANOVA (**a**–**g**, **i**). Significance of difference was analyzed by Sidak's multiple comparisons test (**a**–**g**, **i**). * *p* < 0.05, **p < 0.01, ***p < 0.001, ****p < 0.0001

As discussed in the text above, we postulated that MUC1 possibly affects radiotherapy-induced ferroptosis of ESCC cells. To validate this conjecture, we analyzed the cell damage in Co^+^ ESCC cells under high-dose (8 Gy) irradiation and MUC1 and SIGLEC9 alterations. Importantly, MUC1 overexpression in ESCC cells reduced the radiosensitivity of cells, as manifested by increased DNA replication and reduced cell necrosis (Fig. 8a, b), as well as enhanced cell migration and invasion (Fig. 8c, d). However, the MUC1 overexpression-mediated radioresistance was blocked upon SIGLEC9 blockade in TAMs (Fig. 8a–d).

Overexpression of MUC1 was found to suppress ROS but elevate GPX4 in the 8 Gy-treated Co^+^ ESCC cells, indicating significantly suppressed ferroptosis. Again, the suppressive effect of MUC1 on cell ferroptosis was blocked when SIGLEC9 was blocked in the TAMs (Fig. 8e, f).

The mechanism responsible for MUC1-mediated ferroptosis was further explored. Interaction of MUC1-expression malignant cells and SIGLEC9-expressing immune cells in TME has been reported to promote the recruitment and nuclear translocation of β-catenin [21]. Activated β-catenin pathway can suppress cell ferroptosis by manipulating GPX4 expression and glutathione metabolism [22]. The TOP/FOP Flash assay was performed and revealed that MUC1 promoted the activity of β-catenin pathway in ESCC cells, but this promotion was ineffective again when SIGLEC9 in the co-cultured TAMs was blocked (Fig. 8g). The direct binding between MUC1 and β-catenin was further validated by the Co-IP assay (Fig. 8h). The WB analysis further showed that the protein level of nuclear β-catenin was elevated by oe-MUC1. However, this elevation was diminished again upon SIGLEC9 blockade in TAMs (Fig. 8i).

### Suppression of SIGLEC9-mediated TAM re-education inhibits nuclear translocation of MUC1-β-catenin in ESCC cells to reduce radioresistance

Now that SIGLEC9 suppression in TAMs has been found to conquer the radioresistance of ESCC cells in vitro, we further focused on the roles of the concerned molecules in vivo. ESCC cells were injected into mice subcutaneously or via tail vein to induce xenograft tumors. The mice were exposed to high-dose (8 Gy) radiotherapy and treated with the neutralizing antibody of SIGLECE (paralogous gene functional equivalent to human SIGLEC9) (Fig S2A).

In subcutaneous xenograft tumor models, we found that radiotherapy significantly reduced the tumor weight. Treatment of anti-SIGLECE further improved the tumor-suppressive effect (Fig S2B). The IHC assay showed that the radiotherapy reduced the expression of Ki-67, a tumor growth marker, in tumor tissues; however, the infiltration of CD8^+^ T cells in tumor tissues was suppressed as well. Of note, the anti-SIGLECE treatment further reduced Ki-67 expression in the tumor tissues, and it increased the CD8^+^ T cell infiltration and reduced the expression of SIGLECE and CD206 (Fig S2C-F). The tumor tissues were collected and digested to cell suspension. The WB analysis showed that the protein levels of MUC1 and β-catenin in the nuclei were significantly increased by radiotherapy but decreased by Anti-SIGLECE treatment (Fig S2G). The ELISA results showed that the ROS level was increased, whereas the GPX4 level was decreased in the collected tumor cells after radiotherapy (Fig S2H-I). These results indicated that although nuclear MUC1 and β-catenin were upregulated, they could not completely block the oxidative stress and ferroptosis of tumor tissues induced by radiotherapy. Moreover, the oxidative stress and ferroptosis of cells were further promoted by Anti-SIGLECE treatment (Fig S2H-I). In the tumor metastasis model, we observed that the infiltration of metastatic tumor tissues in mouse lung tissues was suppressed by radiotherapy, and this effect was further strengthened by the SIGLECE antibody treatment as well (Fig S2J).

## Discussion

Upon irradiation, inflammatory cytokines are secreted and immune cells are recruited to activate the immune response and attack tumors; however, radioresistant immunosuppressive cells are also increased within TME, which governs the balance between the activation or suppression of immune system [12]. In this work, we identified that the LINC01004-SPI1 axis leads to SIGLEC9 activation, which engages MUC1 to trigger the formation of immunosuppressive TME and radioresistance in ESCC.

First, we identified SIGLEC9 as a significantly elevated gene in ESCC tissues after radiotherapy in ESCC tissues via the GEO dataset GSE137867. SIGLEC9 is an immune suppressor expressed on several types of immune cells such as granulocytes, B cells, natural killer cells, monocytes, and monocyte-derived macrophages [7]. Here, we performed single-cell analysis, IHC, and immunofluorescence staining and identified that SIGLEC9 was mainly expressed on macrophages in ESCC tissues. This is partly in line with the previous findings that SIGLEC9 is mainly expressed on macrophages in lung cancer sections [8] and glioma [28]. Moreover, we identified that high (> 4 Gy) or low doses (< 1 Gy) of irradiation promoted, whereas the moderate doses (1–4 Gy) of irradiation reduced M2 polarization of TAMs. Indeed, radiotherapy has a dose-dependent effect on the reprogramming of macrophages through several possible mechanisms such as reactive ROS, DNA damage, ataxia telangiectasia mutated (ATM), activation of nuclear factor-kappa B (NF-κB) and mitogen-activated protein kinase pathways [29]. For instance, low dose of X-ray irradiation (< 1 Gy) suppressed the nuclear translocation of NF-κB p65 and IL-1β secretion in M1 macrophages to induce an M2 shift [30, 31]. High dose of irradiation (3 × 20 Gy) induced M2 reprogramming of macrophages by promoting the formation of NFκB p50 heterodimers that are associated with transcriptional activation of IL-10 (M2) but transcriptional suppression of TNFα (M1) [32]. Radiotherapy can affect the accumulation of radicals including ROS that dynamically influence macrophage polarization, with moderate doses of irradiation reportedly elevating ROS production and promoting M1 polarization of macrophages through nicotinamide adenine dinucleotide phosphate oxidase 2-dependent activation of ATM [29]. Meanwhile, ROS is correlated with NFκB activation [33]. Radiotherapy can also induce DNA damage directly or indirectly through ROS-dependent interactions with DNA, which recruits ATM kinase and initiates the DNA repair machinery to modulate macrophage reprograming [29]. Moreover, the work by Seifert et al*.* demonstrates that high dose of radiotherapy directly induces the expression of macrophage-colony stimulation factor, which might represent a primary mechanism driving TAM infiltration and preferential M2 polarization [34]. Of note, we identified the M2 polarization of TAMs upon irradiation showed a positive correlation with the SIGLEC9 expression. In agreement with this, SIGLEC9 was found to upregulate PD-L1 and IL-10 levels in TAMs, namely inducing an immunosuppressive condition [13]. In patients with glioma, high SIGLEC9 expression was detected in high-grade tumors, which was positively linked to the M2 skewing of TAMs [28]. M2 TAMs are one of the most radioresistant cells [12]. Given the fact the SIGLEC9 antibody treatment blocked radiotherapy-induced M2 polarization of TAMs and growth of the co-cultured ESCC cells, we opine that low or high doses of radiotherapy upregulate SIGLEC9 to induce M2 polarization of macrophages, therefore promoting radioresistance in ESCC.

When exploring the underpinnings responsible for aberrant SIGLEC9 expression in the TAMs, we obtained a LINC01004-SPI1 axis that activates SIGLEC9 transcription through comprehensive bioinformatics analyses. SPI1 is a transcription factor and a key regulator of signal communication in the immune system and is critical for the development of myeloid cells and lymphocytes [35]. Disruption of SPI1 has been associated with defects in macrophages, neutrophils, and lymphocytes [36]. Coincidentally, upregulation of SPI1 has been reported in ESCC and correlated with M2 macrophage maintenance [37]. The transcription regulation of SPI1 on the downstream targets in several cancer cases has been reported to involve lncRNAs [26, 27]. The lncRNAs are over 200-nt noncoding RNAs fulfilling key functions in governing chromatin dynamics, gene expression, growth, differentiation, and development of cancer [38]. Moreover, abnormal expression of lncRNAs in TME may affect the proliferation, dissemination, and treatment resistance of cancer cells via multiple mechanisms [39], one of which is that they may bind to transcription factors directly to manipulate gene expression [40]. Here, we performed immunoprecipitation and luciferase assays and confirmed that LINC01004 recruited SPI1 in the nucleus of macrophages, in which SPI1 activated SIGLEC9 transcription. The indispensability of the LINC01004-SPI1 axis in SIGLEC9-mediated M2 TAM reprogramming and radioresistance was confirmed by rescue experiments where knockdown of either LINC01004 or SPI1 led to an M1 shift of TAMs and consequently alleviated the immunosuppressive TME and radioresistance in ESCC.

Cancer cell-expressed MUC1 can be modified by multiple short, sialylated O-linked glycans, which can engage SIGLEC-9 and consequently induce calcium flux and MEK-ERK activation, leading to TAM-like phenotype [17]. We, therefore, analyzed the engagement of MUC1 with SIGLEC9 in irradiation-induced TME alteration and radiotherapy. Of note, we found that low or high doses of irradiation upregulated, whereas moderate doses reduced MUC1 expression in macrophages, which showed a similar trend as SIGLEC9. Likewise, increased MUC1 expression was detected in human colon carcinoma HT-29 cells after 6 Gy of X-irradiation [41]. Compelling evidence demonstrates that MUC1 is closely linked to radioresistance in tumors [42, 43]. As radiotherapy mainly induces ferroptosis to diminish cancer cells [19], production of anti-oxidative molecules, such as manganese superoxide dismutase that scavenges superoxide (O_2_^−^) ions and reduces ROS or reactive nitrogen species, is a major mechanism of radioresistance [29]. Thereafter, we induced overexpression of MUC1 in ESCC cells and found it conferred to the immunosuppressive TME and growth and mobility of cells, and it protected cancer cells from radiotherapy-induced ferroptosis by inducing nuclear translocation of β-catenin. β-catenin can upregulate GPX4 expression [22] to protect against ferroptosis. More specifically, a recent study by Wang et al*.* demonstrates that the β-catenin/TCF4 transcription complex directly binds to the GPX4 promoter to induce its upregulation and the consequent ferroptosis resistance [44]. SIGLEC-9-positive immune cells were associated with several types of MUC1-positive tumor cells. When MUC1-expressing cells ligate with recombinant soluble SIGLEC-9, β-catenin can be recruited to the MUC1 C-terminal domain, which inhibits the β-catenin phosphorylation by GSK-3β and consequently enhances its stabilization and nuclear translocation [21]. Therefore, further induced SIGLEC9 inhibition in TAMs and had them co-cultured with the MUC1-overexpressing ESCC cells. Noteworthily, the M2 skewing of TAMs, radioresistance, activity and ferroptosis resistance of ESCC cells, and the nuclear translocation of β-catenin mediated by MUC1 were blocked upon SIGLEC9 inhibition, signifying the indispensable role of SIGLEC9 in the MUC1-mediated radioresistance and immunosuppressive TME formation.

Taken together, this study demonstrates that the LINC01044-SPI1 axis mediates transcriptional activation of SIGLEC9 in TAMs after high doses of radiotherapy. SIGLEC9 interacts with MUC1, which therefore induces M2 polarization of TAMs, formation of the immunosuppressive TME, nuclear translocation of β-catenin, and ferroptosis resistance of ESCC cells (Fig S3). Therefore, specific inhibition of SIGLEC9 may help overcome the radioresistance in ESCC treatment. However, macrophage reprogramming is a highly complex process during cancer progression and following anti-cancer treatments, which involves interaction of a multitude of molecules. The exact mechanisms linking specific irradiation doses to macrophage skewing, and the causative factors responsible for the activation of the LINC01044-SPI1 axis require further investigations. We would like to focus on these issues in the future researches.

## Supplementary Information

Below is the link to the electronic supplementary material.

Supplementary file1 (DOCX 18 KB)

Supplementary file2 (DOCX 12 KB)

Supplementary file3 (DOCX 678 KB)

## Data Availability

All data included in this study are available upon request by contact with the corresponding author.
