# Supplementary material for: LINC01004-SPI1 axis-activated SIGLEC9 in tumor-associated macrophages induces radioresistance and the formation of immunosuppressive tumor microenvironment in esophageal squamous cell carcinoma
Source: Cancer Immunol Immunother. 2023 Jan 23;72(6):1835–51. doi: 10.1007/s00262-022-03364-5 (PMC10198857; doi:10.1007/s00262-022-03364-5)
Supplement: Supplementary file 1 — Supplementary file1 (DOCX 18 KB) [file 262_2022_3364_MOESM1_ESM.docx]

**Supplementary Materials and Methods**

**Immunohistochemistry (IHC)**

Paraffin-embedded tumor tissue sections (5 μm) were dewaxed, treated with H_2_O_2_ for endogenous peroxidase blockade, treated with Tris/EDTA buffer (pH 9.0) for heat-mediated antigen retrieval, and blocked with 5% bovine serum albumin (BSA) at room temperature (20-25℃) for 20 min. Thereafter, the sections were reacted with the antibodies including CD14 (1:200, ab182032, Abcam Inc., Cambridge, MA, USA), MUC1 (1:200, ab109185, Abcam), SIGLEC9 (1:200, ab197981, Abcam), SIGLECE (1:200, AF5806, Novus Biologicals Inc., Littleton, CO, USA), CD8 (1:2,000, ab217344, Abcam), CD206 (1:200, ab64693, Abcam), and Ki67 (1:1,000, ab15580, Abcam) at 4℃ overnight, and then with goat ant-rabbit immunoglobulin G (IgG) H&L (HRP) (1:1,000, ab6721, Abcam) or goat anti-mouse IgG H&L (HRP) (1:1,000, ab205719, Abcam) at room temperature for 2 h. DAB was used for color development, and the nuclei were counter-stained with hematoxylin. The sections were then sealed for microscopy observation to analyze the positive staining (brownish staining). The number of positive cells (protein localized in nucleus) or the area of positive staining (protein localized in cytoplasm) was analyzed by Image J.

**Double-label immunofluorescence**

The 5-μm tumor tissue sections were penetrated with 0.25% Triton X-100 for 10 min, and blocked with 1% BSA for 1 h. Afterward, the sections were incubated with the SIGLEC9 antibody (1:200, PA5-115900, Thermo Fisher Scientific) at 4℃ overnight and Alexa Fluor 647-labeled goat anti-rabbit IgG (1:1,000, A0468, Beyotime Biotechnology Co., Ltd., Shanghai, China) at room temperature in the dark for 1 h. Later, the sections were further reacted with anti-CD14 (1:200, #MA1-33348, Thermo Fisher Scientific) at 4℃ overnight and then with Alexa Fluor 488-labeled goat anti-mouse IgG (1:1,000, A0428, Beyotime). The sections were sealed by neutral resin and then observed under the confocal fluorescence microscopy (Zeiss Inc, AG, Oberkochen, Germany) to analyze the co-localization of SIGLEC9 and CD14.

The macrophages (see details below) were fixed with formaldehyde for 10 min and then penetrated with 1% Triton X-100 for 5 min. The cells were incubated with Cy3-labeled LINC01004-specific fluorescence probe at 4°C overnight for *in situ* hybridization. The probe was rinsed away with phosphate-buffered saline (PBS), and then the cells were reacted with anti-SPI1 (1:1,000, ab88082, Abcam) at 4°C overnight, and then with Alexa Fluor 488-labeled goat anti-mouse IgG (1:1,000, A0428, Beyotime) at room temperature in the dark for 2 h. The nuclei were stained with DAPI. The cell slides were sealed, and the co-localization of SPI1 and LINC01004 was observed under the fluorescence microscopy.

**Cells**

CD14^+^ monocytes and CD8^+^ T cells were isolated from human peripheral blood samples using the Dynabeads™ FlowComp™ human CD14 and Dynabeads™ CD8 isolation kits (both from Invitrogen, Thermo Fisher Scientific, Rockford, IL, USA). ESCC cell lines TE-1 (CL-0231) and KYSE-30 (CL-0577) with short tandem repeat authentication were procured from Procell Life Science & Technology Co., Ltd. (Wuhan, Hubei, China). The cells were cultured in Roswell Park Memorial Institute (RPMI)-1640 added with 1% penicillin/streptomycin and 10% fetal bovine serum (FBS) at 37℃ with 5% CO_2_.

The isolated monocytes were cultured in RPMI-1640 containing 1% penicillin/streptomycin, 10% FBS, 1% L-glutamine, and 100 ng/mL recombinant macrophage-colony stimulating factor at 37℃ with 5% CO_2_ for 7 d to differentiate into macrophages. Dynabeads™ Human T-Activator CD3/CD28 (Gibco, Thermo Fisher Scientific) was used to induce activation and amplification of human T cells.

The macrophages were cultured with ESCC cells or T cells at 1:3 or 1:1, respectively. After co-culture, the macrophages in the co-culture system were isolated by the Dynabeads™ FlowComp™ human CD14 kit.

**Cell treatment**

The DNA overexpressing plasmid of MUC1, the short hairpin (sh) RNA (mammal shRNA interfering vector poggyBac) of SPI1, and the negative control (NC) vectors were procured from VectorBuilder Inc. (Guangzhou, Guangdong, China) and transfected into cells Lipofectamine 3000 (Thermo Fisher Scientific). Antisense oligonucleotides (ASOs) used for LINC01004 knockdown were procured from RiboBio Co., Ltd. (Guangdong, China) and transfected into cells using the riboFECT™ CP Transfection Reagent (RiboBio). Cells were used for subsequent experiments at 48 h after transfection. The macrophages were pre-incubated with the neutralizing monoclonal antibody of SIGLEC9 (MAB1139-500, Novus Biologicals) for 24 h to block SIGLEC9. The isotype IgG was used as control, with the final concentration of 0.075 μg/mL. The shRNA and ASO sequences are listed below: sh-SPI1 1#: 5ʹ-AGCGAGTTCGAGAGCTTCGCCGAGAACAACTTCACGGAGCT-3ʹ; sh-SPI1 2#: 5ʹ-AAGACCTGGTGCCCTATGACACGGATCTATACCAACGCCAA-3ʹ; sh-SPI1 3#: 5ʹ-AGGGCAACCGCAAGAAGATGACCTACCAGAAGATGGCGCGC-3ʹ. ASO-LINC1#: ACTGATTTGCAAGGCCCCGGGT; ASO-LINC2#: CTCCATAAGTGTGTTCCATCTG; ASO-LINC 3#: TGTTTCAAGAACGCCTCAATAT.

**Flow cytometry**

The isolated macrophages were resuspended in PBS and adjusted to 1 × 10^7^ cells/mL. Thereafter, 100 μL cell suspension was incubated with 5 μL receptor blocking solution (Cat #422301, Biolegend, San Diego, CA, USA) at room temperature for 10 min, and then with 5 μL FITC-conjugated CD11C (Cat #337213, Biolegend), PE-conjugated CD163 (Cat #333605, Biolegend) at 4℃ for 30 min. Right after that, the cells were examined on the CytoFLEX flow cytometer (Beckman Coulter, Inc., Chaska, MN, USA) and analyzed by FlowJo software (10.0.7).

For carboxyfluorescein diacetate succinimidyl ester (CFSE) staining, the T cells were resuspended in PBS and adjusted to 2 × 10^7^ cells/mL. Next, 0.5 μL CFSE solution (AbMole BioScience, Houston, TX, USA) was added to each 1 mL cell suspension and incubated at 37℃ for 10 min till a final concentration of 1 μM. CFSE-precoated T cells were co-cultured with macrophages for 5 d. The fluorescence intensity of CFSE was analyzed by flow cytometry, and the portion of proliferating T cells was analyzed by FlowJo.

**Cell necrosis detection**

The survival and necrosis of ESCC cells was analyzed using the 7-AAD cell viability detection kit (C1053S, Beyotime) and the Calcein AM cell viability detection kit (C2013S, Beyotime). In short, treated ESCC cells were seeded in 96-well plates at 5,000 cells/well. The cells were incubated with 100 μL Calcein AM working solution at 37℃ in the dark for 30 min, and then with 100 μL 7-AAD working solution for 10 min. The cells were observed under the confocal fluorescence microscopy to analyze the portion of necrotic cells.

**5-ethynyl-2’-deoxyuridine (EdU) labeling assay**

The DNA damage in ESCC cells was analyzed using the Cell-Light EdU Apollo643 *In Vitro* Kit (RiboBio). The ESCC cells were seeded in 96-well plates at 4,000 cells per well. Each well was loaded with 100 μL EdU reagent (50 μM) for 2 h. Afterward, the cells were fixed for 30 min, penetrated with 0.5% TritonX-100 for 10 min, and reacted with 1× Apollo® reaction reagent at room temperature in the dark for 30 min. Later, the cells were reacted with 1× Hoechst 33342 solution for 30 min of nuclear staining. Thereafter, the cells were observed under the microscopy to analyze the portion of EdU-positive cells.

**Transwell assays**

The invasion and migration capacities of ESCC cells were analyzed using Transwell chambers. The ESCC cells were resuspended in serum-free medium and loaded into the 24-well Transwell chambers pre-coated with Matrigel (Matrigel pre-coating for invasion detection only). The basolateral chambers were filled with 600 μL culture medium containing 10% FBS. After 24 h of incubation, the cells were fixed. Cells remained on the upper side were discarded, and those invaded or migrated to the lower side were stained with 0.1% crystal violet for 10 min. The cells were observed under microscopy. The mean value of cell counts in five random fields was calculated.

**Enzyme-linked immunosorbent assay (ELISA) and oxidative stress examination**

The concentrations of transforming growth factor beta 1 (TGF-β1) and GPX4 were analyzed by TGF-β1 (ab100647, Abcam) and GPX4 (KTE63061, Abbkine Scientific Co., Ltd., Wuhan, Hubei, China) ELISA kits. Specific antibodies of TGF-β1 and GPX4 were used to analyze the existence of TGF-β1 and GPX4 in the samples, and the HRP-conjugated streptavidin to detect the reaction signal. The TMB substrate solution was used for color development. The optical density at 450 nm was read to detect the expression of the concerned factors. The ROS in cells was detected using the H2DCFDA (cell permeable fluorescence probe) of the ROS detection kit (K936, BioVision, Milpitas, CA, USA). The mean fluorescence intensity was observed and calculated by the fluorescence microscope (Ex/Em 495/529 nm).

**Western blot (WB) analysis**

Whole cellular protein or nuclear protein from cells was extracted using the RIPA lysis buffer (R0020, Solarbio Science & Technology Co., Ltd., Beijing, China) or the EpiQuik Nuclear Extraction Kit (OP-0002-1, Epigentek, Farmingdale, NY, USA), respectively. After protein quantification using the Pierce^TM^ BCA kit (Thermo Fisher Scientific), equal amounts of protein sample were separated by SDS-PAGE and loaded on polyvinylidene fluoride membranes. Afterwards, the membranes were blocked with 3% BSA for 1 h and then incubated with diluted antibodies at 4°C overnight and with the secondary antibody at room temperature for 2 h. The signals were examined using the ECL kit (Beyotime) and quantified using Image J. GAPDH was used as the endogenous loading for whole cellular protein, while Lamin B1 as the loading for nuclear protein. The primary antibodies included SPI1 (1:2,000, ab88082, Abcam), MUC1 (1:1,000, ab109185, Abcam), SIGLEC9 (1:2,000, ab197981, Abcam), β-catenin (1:5,000, ab32572, Abcam), GAPDH (1:1,000, #5174, Cell Signaling Technology [CST], Beverly, MA, USA), and Lamin B1 (1:2,000, ab16048, Abcam). The secondary antibody used was IgG H&L (HRP) (1:2,000, ab6721, Abcam).

**Chromatin immunoprecipitation (ChIP)**

Macrophages (1 × 10^7^) were crosslinked in 1% paraformaldehyde and neutralized with glycine. Thereafter, the cells were lysed and ultrasonicated for DNA truncation. The lysates were then reacted with anti-SPI1 (#2266, CST) or normal rabbit IgG (#2729, CST) at 4°C overnight and added with Dynabeads protein A/G magnetic beads (Invitrogen) for 4 h to collect the IP complexes. The DNA was eluted and purified, and the precipitated DNA was quantified by qPCR analysis using the specific SIGLEC9 promoter primer.

**Luciferase reporter assay**

The binding region between SPI1 and SIGLEC9 promoter was obtained from JASPAR (http://jaspar.genereg.net/). The SIGLEC9 promoter sequence containing the putative binding region was inserted into the pGL3-Basic vector (Promega Corporation, Madison, WI, USA) to construct luciferase reporter vector of the SIGLEC9 promoter. The vector was transfected into TAMs along with sh-SPI1 or sh-NC, followed by radiotherapy or not. After 48 h, the luciferase activity in cells was analyzed by the dual luciferase reporter gene kit (Beyotime).

The TOP Flash (D2501) and FOP Flash (D2503) used to analyze the transcriptional activity of β-catenin were procured from Beyotime and transfected into ESCC cells using Lipofectamine. The luciferase activity in cells was analyzed 24 h later.

**RNA immunoprecipitation (RIP) and** **Co-immunoprecipitation (Co-IP)**

Cells were lysed in RIPA buffer. The supernatant was collected and incubated with anti-SPI1 (1:100, #2266, CST) or anti-MUC1 (1:20, ab109185, Abcam) at 4°C overnight, with the isotype IgG used as control. The antibody-conjugated samples were incubated with protein A Sepharose (Sigma-Aldrich, Merck KGaA, Darmstadt, Germany) at 4°C for 2 h. The samples were then eluted with the lysis buffer. For the complexes precipitated by anti-SPI1, the RNA was extracted from the elution solution, and the enrichment of LINC01004 was detected by reverse transcription and qPCR analysis. For the complexes precipitated by anti-MUC1, the protein was extracted, and the enrichment of β-catenin protein was analyzed by WB analysis.

**Animals**

A total of 128 immune active female C57BL/6 mice (6 weeks old) were procured from SLAC Laboratory Animal Co., Ltd. (Shanghai, China) and allocated into the following four groups: RT^–^ group (mice without radiotherapy), RT^+^ group (mice received radiotherapy), RT^+^ + IgG group (mice received radiotherapy and IgG treatment), and RT^+^ + Anti-SIGECE group (mice received radiotherapy and Anti-SIGECE treatment), n = 32 in each.

ESCC cells (TE-1 and KYSE-30) resuspended in PBS were mixed with Matrigel and subcutaneously injected into the mice (1 × 10^6^ cells per mouse) at the right flank to induce subcutaneous tumors. When the tumor size reached around 150 mm^3^, the tumor site was locally exposed to irradiation (2 Gy/d for consecutive 4 d). For antibody injection, the mice were injected with IgG or Anti-SIGECE on day 1, 7, or 14 after the first irradiation exposure. After 28 d, the mice were euthanized via overdosed barbiturate (150 mg/kg). The subcutaneous tumors were collected for IHC. Another group of ESCC cells were injected into mice via tail vein (2 × 10^6^ cells per mouse). Alike, the mice were subjected to radiotherapy and antibody injection. On day 35, the mice were euthanized, and the lung tissues were collected for hematoxylin and eosin (HE) staining.
