# Supplementary material for: LINC01004-SPI1 axis-activated SIGLEC9 in tumor-associated macrophages induces radioresistance and the formation of immunosuppressive tumor microenvironment in esophageal squamous cell carcinoma
Source: Cancer Immunol Immunother. 2023 Jan 23;72(6):1835–51. doi: 10.1007/s00262-022-03364-5 (PMC10198857; doi:10.1007/s00262-022-03364-5)
Supplement: Supplementary file 2 — Supplementary file2 (DOCX 12 KB) [file 262_2022_3364_MOESM2_ESM.docx]

**Table S1** Primers used for qPCR analysis

| Gene | Forward sequence (5ʹ-3ʹ) | Reverse sequence (5ʹ -3ʹ) |
| --- | --- | --- |
| IL-10 (NM_000572) | TCTCCGAGATGCCTTCAGCAGA | TCAGACAAGGCTTGGCAACCCA |
| PD-L1 (NM_014143) | TGCCGACTACAAGCGAATTACTG | CTGCTTGTCCAGATGACTTCGG |
| TNF-α (NM_000594) | CTCTTCTGCCTGCTGCACTTTG | ATGGGCTACAGGCTTGTCACTC |
| IL-12 (NM_002187) | GACATTCTGCGTTCAGGTCCAG | CATTTTTGCGGCAGATGACCGTG |
| SIGLEC9 (NM_014441) | CCACGAACAAGACCGTCCATCT | TCTGGGAGTGACAGAGATGAGC |
| SPI1 (NM_003120) | GACACGGATCTATACCAACGCC | CCGTGAAGTTGTTCTCGGCGAA |
| SIGLEC9 promoter | ACCTCTAACCCCAGACATGC | GTAAATCCAGCCATGCGAGG |
| LINC01004 (NR_039981) | CCATTGGCTTGCCTAACAGC | TGACTCTTGGCTAAGCTGGC |
| MUC1 (NM_002456) | CCTACCATCCTATGAGCGAGTAC | GCTGGGTTTGTGTAAGAGAGGC |
| GAPDH (NM_002046) | GTCTCCTCTGACTTCAACAGCG | ACCACCCTGTTGCTGTAGCCAA |

Note: qPCR, quantitative polymerase chain reaction; IL-10, interleukin 10; PD-L1, programmed cell death 1 ligand 1; TNF-α, tumor necrosis factor-α; IL-12, interleukin 12; SIGLEC9, sialic acid binding Ig like lectin 9; SPI1, Spi-1 proto-oncogene; LINC01004, long intergenic non-protein coding RNA 1004; MUC1, mucin 1; GAPDH, glyceraldehyde-3-phosphate dehydrogenase
