# Supplementary material for: LINC01004-SPI1 axis-activated SIGLEC9 in tumor-associated macrophages induces radioresistance and the formation of immunosuppressive tumor microenvironment in esophageal squamous cell carcinoma
Source: Cancer Immunol Immunother. 2023 Jan 23;72(6):1835–51. doi: 10.1007/s00262-022-03364-5 (PMC10198857; doi:10.1007/s00262-022-03364-5)
Supplement: Supplementary file 3 — Supplementary file3 (DOCX 678 KB) [file 262_2022_3364_MOESM3_ESM.docx]

**Fig S1**

**
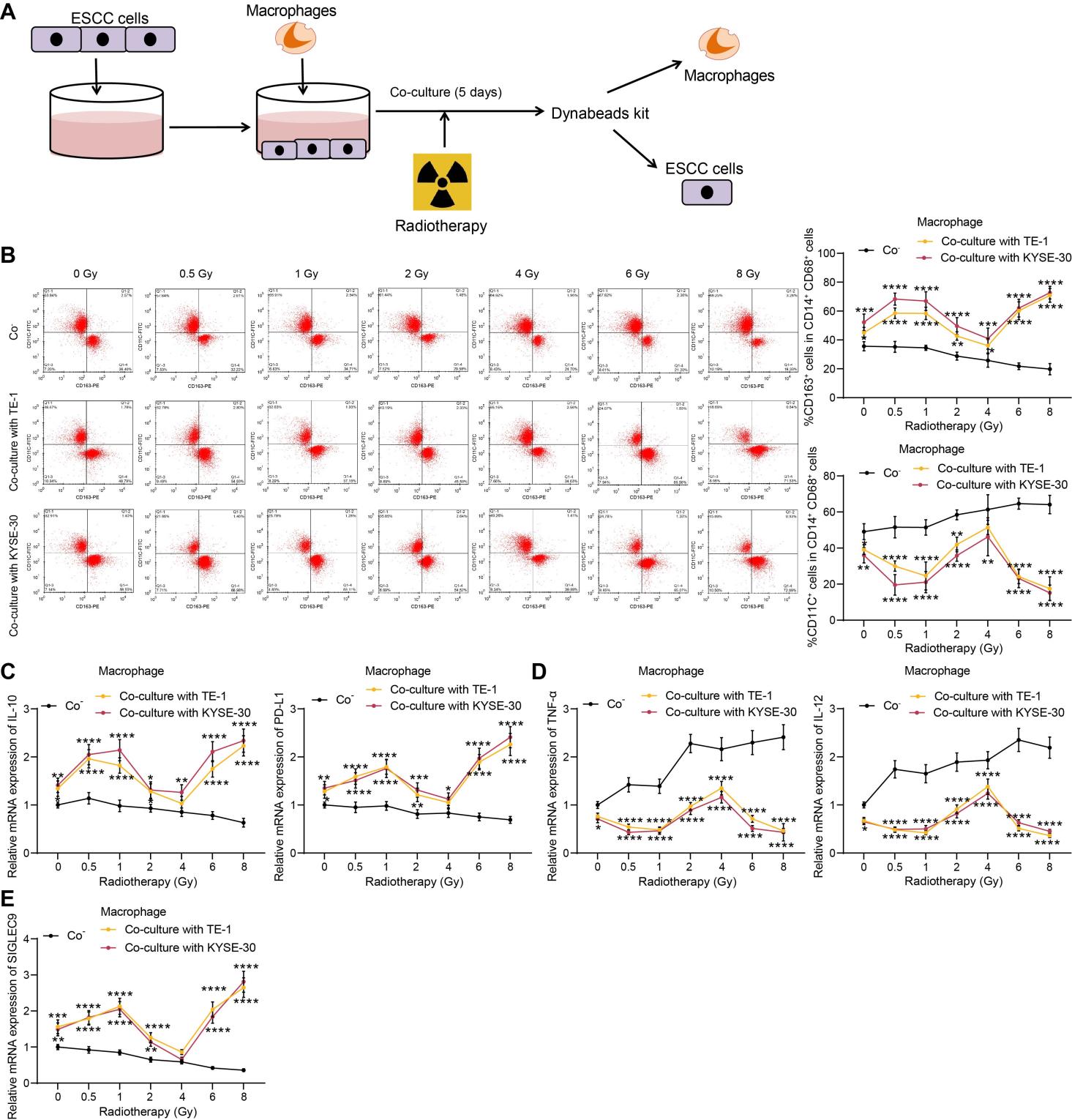
**

**Fig S1** Radiotherapy induces phenotype change of TAMs. A, a sketch map for the co-culture system of macrophages and ESCCs; B, phenotype change of macrophages analyzed by flow cytometry; C-D, expression of M2 phenotype markers IL-10 and PD-L1 (C) and M1 phenotype markers TNF-α and IL-12 (D) in macrophages analyzed by qPCR analysis; E, expression of SIGLEC9 in macrophages determined by qPCR analysis. Differences of the normally distributed data between groups were analyzed by two-way ANOVA (B-E). **p* < 0.05, ***p* < 0.01, ****p* < 0.001, *****p* < 0.0001.

**Fig S2**

**
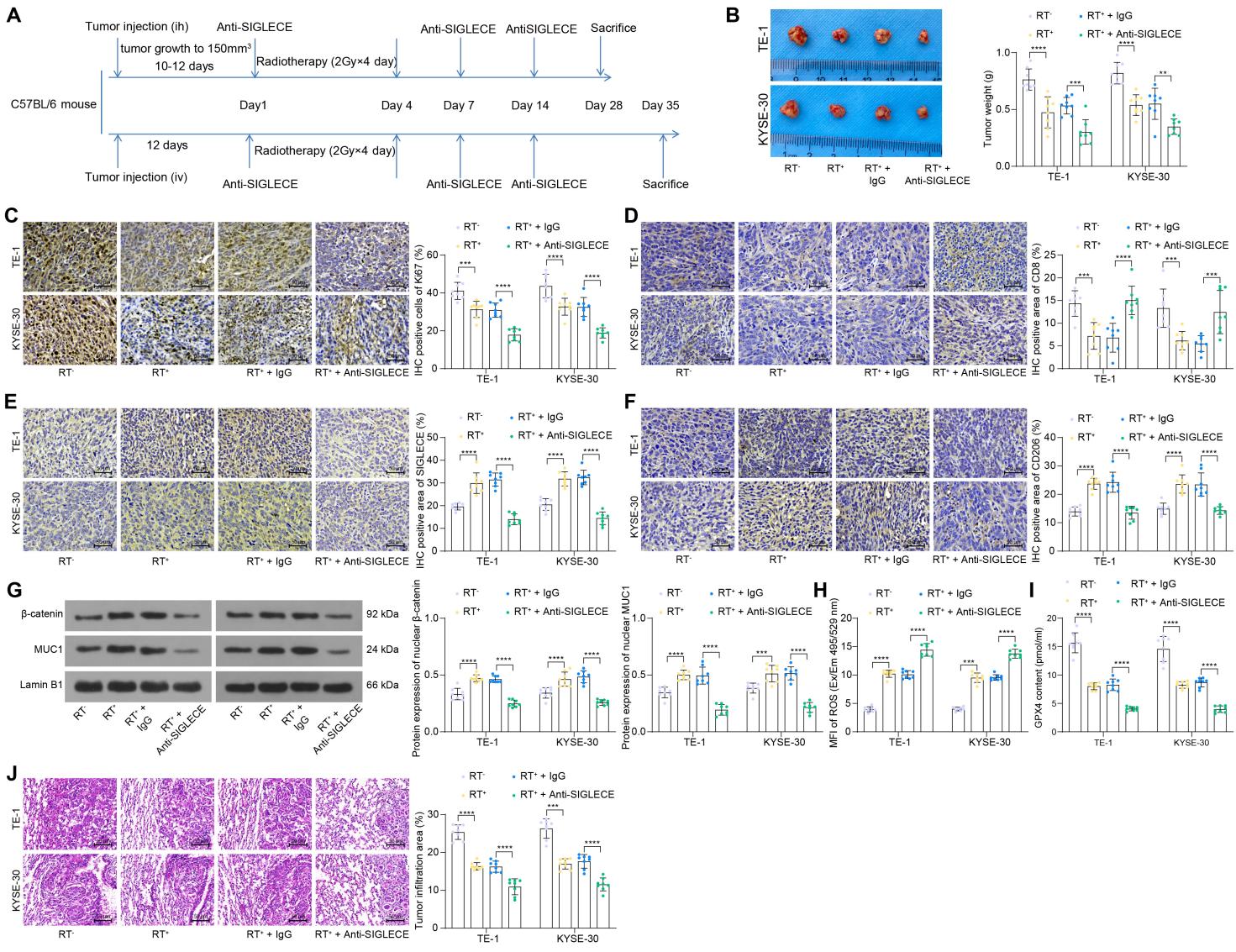
**

**Fig S2** Suppression of SIGLEC9-mediated TAM re-education inhibits nuclear translocation of MUC1-β-catenin in ESCC cells to reduce radioresistance. A, a sketch map for the animal treatment; B, representative images of the subcutaneous xenograft tumors; C-F, expression of Ki67, CD8, SIGLECE, and CD206 in tumor tissues analyzed by IHC; G, protein levels of MUC1 and β-catenin in the nuclei of tumor cells analyzed by WB analysis; H-I, ROS and GPX4 levels in tumor cells analyzed by ELISA kits; J, tumor infiltration in mouse lung tissues upon tail vein injection of cancer cells analyzed by HE staining. Differences of the normally distributed data between groups were analyzed by two-way ANOVA (B-J). Significance of difference was analyzed by Tukey’s multiple comparisons test (B-J). ***p* < 0.01, ****p* < 0.001, *****p* < 0.0001.

**Fig S3**

**
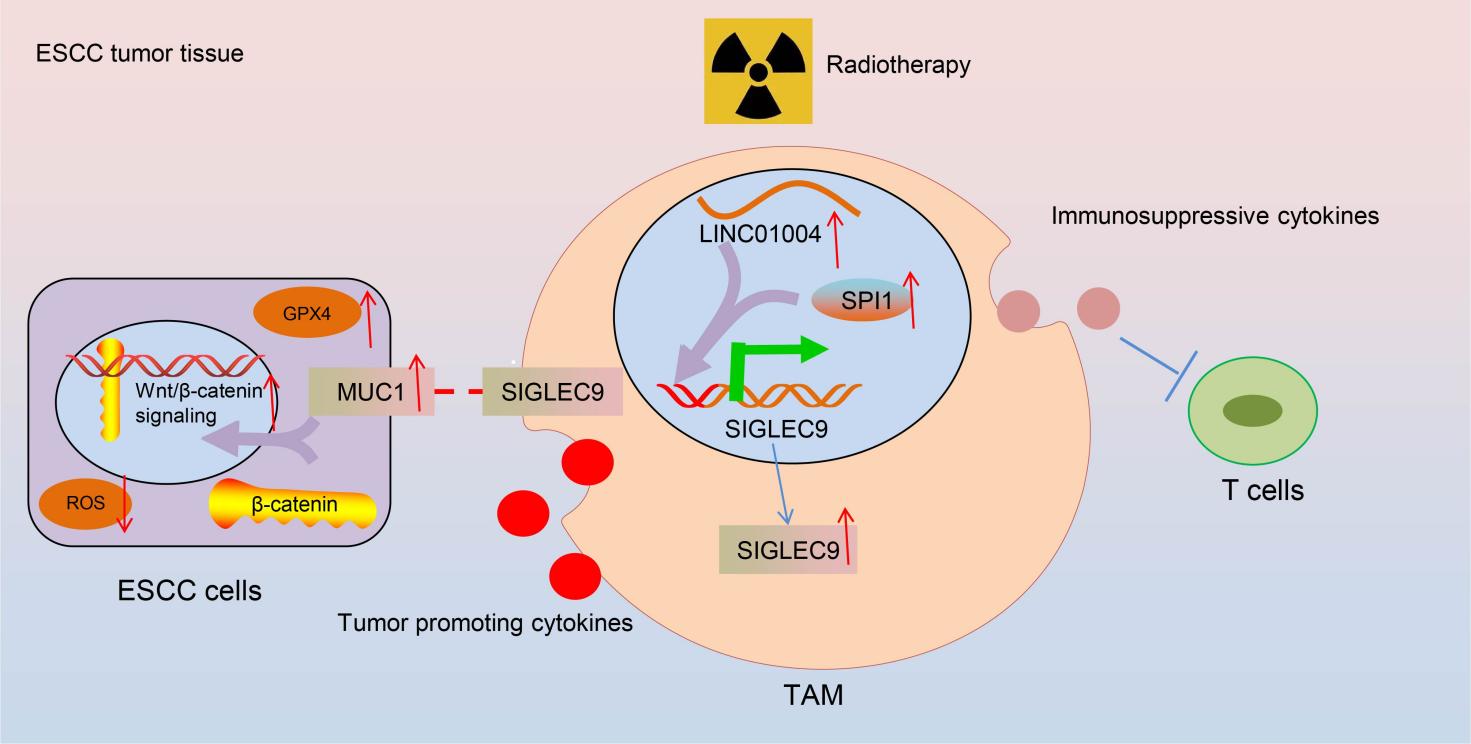
**

**Fig S3** Graphical abstract. Under high doses of radiotherapy**,** the LINC01044-SPI1 axis mediates transcriptional activation of SIGLEC9 in TAMs, which induces M2 reprogramming of macrophages and suppresses proliferation of active T cell to form an immunosuppressive TME. SIGLEC9 interacts with MUC1 expressed on ESCC cells to induce M2 TAM polarization and radioresistance, and this interaction promotes nuclear translocation of β-catenin to protect ESCC cells from radiotherapy-induced ferroptosis.
